# Observation of spatial nonlinear self-cleaning in a few-mode step-index fiber for special distributions of initial excited modes

**DOI:** 10.1038/s41598-021-03856-x

**Published:** 2021-12-21

**Authors:** Zahra Mohammadzahery, Maryam Jandaghi, Ebrahim Aghayari, Hasan Nabavi

**Affiliations:** Iranian National Center for Laser Science and Technology, Tehran, Iran

**Keywords:** Optics and photonics, Physics

## Abstract

In this paper, we experimentally demonstrate that a nonlinear Kerr effect in suitable coupling conditions can introduce a spatially self-cleaned output beam for a few-mode step-index fiber. The impact of the distribution of the initial excited modes on spatial beam self-cleaning has been demonstrated. It is also shown experimentally that for specific initial conditions, the output spatial pattern of the pulsed laser can be reshaped into the LP_11_ mode due to nonlinear coupling among the propagating modes. Self-cleaning into LP_11_ mode required higher input powers with respect to the power threshold for LP_01_ mode self-cleaning. Our experimental results are in agreement with the results of numerical calculations.

## Introduction

Nonlinear pulse propagation in Multimode fibers (MMFs) has recently attracted a lot of attention in recent years due to their high transmission capacity, especially for very high data rate optical communications^1,2^. Research on MMF has revealed a number of nonlinear spatiotemporal phenomena such as multimode solitons, geometric parametric instability (GPI), supercontinuum (SC) generation, and nonlinear microscopy and endoscopy^3–10^. The input spatially Gaussian beam in MMFs, leads to an output speckled beam pattern due to the generation of a large number of guided transverse modes and this is a major problem in transmitting radiation through these fibers. After a few centimeters of the light propagation inside the fiber, the random coupling of the modes leads to a sever reduction in the quality of beam. In recent studies on nonlinear pulse propagation in MMFs it is demonstrated that the refractive index dependence on light intensity (Kerr-effect), in MMFs can lead to a spatially cleaned output beam which is robust against fiber bending^11–15^. The power threshold for frequency conversion or self-focusing of sub nanosecond to femtosecond pulses propagating in the normal dispersion regime is much higher than that required for nonlinear self-cleaning effect. Most articles in this field, reported nonlinear beam reshaping and the other nonlinear effects in MMFs with a parabolic or nearly parabolic refractive index profile^16–19^. We demonstrated for the first time in our previous work that the Kerr beam self-cleaning effect can be seen in step-index few-mode fiber at higher peak-powers with respect to the power threshold for self-cleaning in graded index (GRIN) fibers^20^. It is obvious that the role of core refractive index profile is very important in nonlinear self-cleaning process, by introducing intermodal dispersion and creation of a periodic self-imaging effect^21^. Kerr beam self-cleaning arises due to quasi-phase matching (QPM) induced by mode auto imagery when the refractive index is parabolic. This imagery can occur in step-index fibers but not with the same efficiency and with a smaller coherence length than GRIN fibers^22^. With few-mode fibers that support fewer modes than MMFs, it is easier to have the QPM condition between modes than highly multimode fibers. In this work, we demonstrate that for creation of self-cleaning effect in few-mode step-index fiber, it is necessary to manage the coupling condition and consequently the initially guided modes fractions because this arrangement affects the self-imaging distance and nonlinear modal couplings. We focus on the effect of the initial modal distributions on Kerr-beam self-cleaning in few-mode step-index fiber. To show that, we employed the second harmonic of a sub-nanosecond microchip pulsed laser propagating in a step-index fiber with a core diameter of 20 μm and a numerical aperture of 0.065 supporting more than 10 spatial modes at 532 nm wavelength. We found that there is a final limitation for the initial propagating mode distribution in order to achieve a proper Kerr-induced refractive index profile and consequently a nonlinear self-cleaned output beam in few-mode step-index fiber. Moreover, at a special input coupling angle, Kerr nonlinear self-cleaning in our considered fiber can reshape the transverse output pattern into the LP_11_ mode. Nonlinear self-cleaning of the LP_11_ mode requires a careful adjustment of the laser beam coupling at the fiber input to prepare a proper power distribution among the guided modes. Numerical simulations are consistent with our experimental results.

## Theory

We utilized the generalized multimode nonlinear Schrödinger equation (GMM-NLSE) to calculate the propagation of all excited modes in few-mode step-index fiber^23^:

1 $$\begin{aligned} \partial_{z} A_{p} \left( {z,t} \right) & = i\left( {\beta_{0}^{p} - \beta_{0} } \right)A_{p} - \left( {\beta_{1}^{p} - \beta_{1} } \right)\frac{{\partial A_{p} }}{\partial t} \\ & \quad + \;\sum\limits_{m \ge 2} {i^{m + 1} \frac{{\beta_{m}^{p} }}{m!}\partial_{t}^{m} A_{p} + i\frac{{n_{2} \omega_{0} }}{c}\left( {1 + \frac{i}{{\omega_{0} }}\partial_{t} } \right)} \sum\limits_{l,m,n} {\left\{ {\left( {1 - f_{R} } \right)S_{plmn}^{k} A_{l} A_{m} A_{n}^{*} } \right.} \\ & \quad \left. { + \;f_{R} A_{l} S_{plmn}^{R} \int\limits_{ - \infty }^{t} {d\tau A_{m} \left( {z,t - \tau } \right)A_{n}^{*} \left( {z,t - \tau } \right)h_{R} \left( \tau \right)} } \right\} \\ \end{aligned}$$

here ${\text{A}}_{{\text{p}}} \left( {{\text{z}},{\text{t}}} \right)$ is *p*th mode envelop, $\beta_{n}^{p}$ is the (*n*-1)th order term in the Taylor series expansion of the wave number for *p*th mode, $S_{plmn}^{k}$ and $S_{plmn}^{R}$ are mode overlap tensors, $f_{R}$ is the fractional contribution of the Raman response to the total nonlinearity (approximately 0.18), and $n_{2}$ is the nonlinear Kerr parameter of Silica. The electromagnetic field inside the core can be expressed as a superposition of all excited modes:

2 $$\vec{E}\left( {r,\varphi ,z} \right) = \mathop \sum \limits_{l,m}^{N} C_{lm} e_{lm} \left( {r,\varphi } \right){\text{exp}}\left( { - i\beta_{lm} z} \right),$$

where summation is taken over all ($l,m$) excited modes with propagating constant $\beta_{lm}$. Spatial self-imaging effect is one of the initial phenomena in the self-cleaning process, which its combination with Kerr nonlinearity will introduce a periodical modulation of the refractive index in the core of the fiber. Self-imaging is actually the reproduction of the input field in some positions inside the fiber where guided modes are in phase and satisfy the following condition^21^:

3 $$\left( {\beta_{01} - \beta_{lm} } \right)z_{s} = m_{lm} 2\pi ,$$

where $m_{lm}$ is an integer number and $z_{s}$ is the self-imaging distance with one period. With the properties of the characteristic equations^24^, the number of guided modes and corresponding propagation constants can be accurately determined by numerical procedures. According to the computations, an imaging period of 1.8 mm is expected for propagation of 532 nm wavelength for a straight alignment of the input beam, which is much smaller than 10-43 mm in a step-index MMF with 50–105 μm core diameter and slightly longer than 1 mm or less for a GRIN MMF. For high-intensity beams, there is an intensity-dependent refractive index in the MMF core, and the self-imaging period changes according to:

4 $$\left( {\beta_{01} \left( I \right) - \beta_{lm} \left( I \right)} \right)z_{I} = m_{lm} 2\pi .$$

On the other hand, there is an additional phase shift $\gamma_{p} P_{p} z$ in the presence of intermodal nonlinear effects, where $\gamma_{p} = \frac{{n_{2} \omega_{0} S_{pppp} }}{c}$ is nonlinear coefficient and $P_{p}$ is the equivalent optical power for the *p*th mode^25^. Consequently self-imaging distance reduces slightly by the amount of Δ:

5 $$\Delta = \frac{{\gamma_{1} P_{1} - \gamma_{p} P_{p} }}{{\left( {\beta_{01} - \beta_{lm} } \right) + \left( {\gamma_{1} P_{1} - \gamma_{p} P_{p} } \right)}}.$$

As it can be seen, by increasing the optical power of the fundamental mode with respect to the higher order modes, there will be a greater reduction in the self-imaging distance. According to our calculations, for 10 kW input peak-power Δ can be increased up to 0.36 mm for different fractions of fundamental mode with respect to the higher order modes. A reduction in the self-imaging distance facilitates nonlinear interactions, leading to a spatially cleaned output beam in a few-mode step-index fiber. On the other hand, as it has explained in Ref^22^, if there is a higher fraction of fundamental mode in the initial distribution of guided modes, an irreversible decoupling of the fundamental mode can be observed, which allows the power to remain in this mode. As it is demonstrated experimentally and numerically in the next sections, there is a limitation of propagating mode fractions on the observation of the self-cleaning effect in few-mode step-index fiber.

## Experiments

In our experiments, we used a 5 m long piece of step-index fiber with a core diameter 20 µm and numerical aperture of 0.065. The signal source for experiments was the second harmonic of a microchip Nd:YAG laser delivering sub nanosecond (450 ps) pulses at 1 kHz repetition rate with 40 µJ maximum pulse energy at 532 nm wavelength. The laser beam was injected into the fiber using a focusing lens (f = 50 mm) controlled by using a 3-axis translation stage. The laser beam passed through a half-wave plate and a polarization cube before being focused within the central axis onto the fiber input face, in order to adjust the input power. Full with at half maximum in intensity (FWHMI) of the laser beam on the input face of the fiber was 10 µm. The fiber carries more than 10 modes at both polarization components at 532 nm wavelength. The optical setup included CCD cameras for field monitoring, a power meter and a spectrum analyzer.

**Figure 1:**
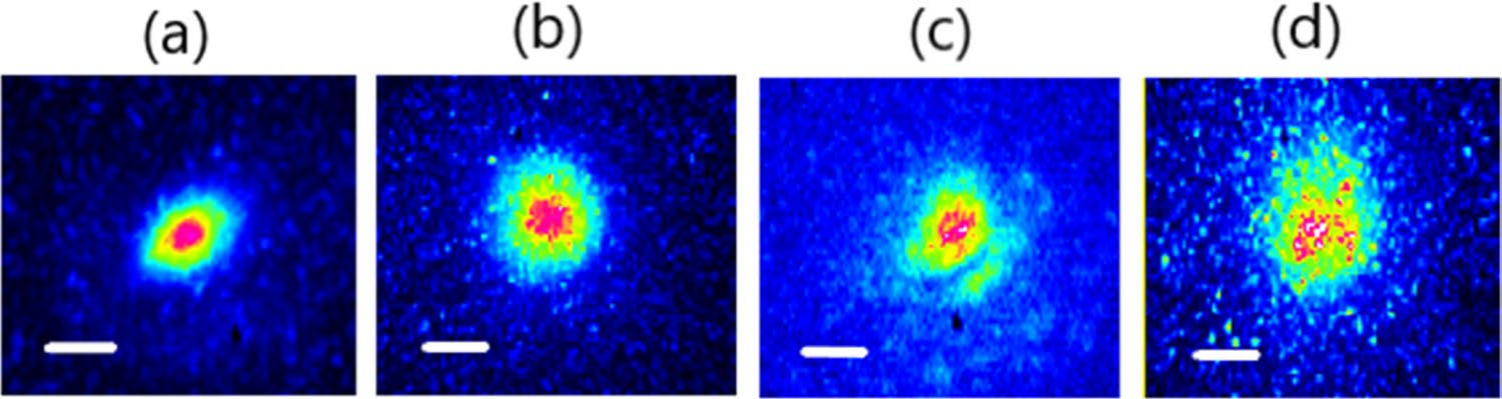
Output beam intensity patterns versus input beam position with respect to the fiber center: (**a**) Δx = 0 μm, (**b**) Δx = 2 μm, (**c**) Δx = 4 μm, (**d**) Δx = 7 μm. Scale bar is 10 μm.

In our previous work, we demonstrated experimentally and numerically that by increasing the injected power from 0.3 kW in the linear regime to 6 kW in the nonlinear regime, the output pattern of the step-index few-mode fiber transfers from a speckled profile to a bell-shaped smooth central beam. Here, in the first series of experiments, we studied the impact of the number of excited modes on the properties of the Kerr-beam self-cleaning process. We injected the input pulse beam into the step-index few-mode fiber by imposing a transverse shift to the entrance of the fiber with respect to the optical lens, so the initial conditions were varied. Figure 1a–d shows the spatial beam profile at the output of the fiber for different input conditions while keeping the guided peak power fixed.

As it can be seen clearly from the figure, the excitation of the few-mode fiber at the central position with 6 propagating modes and a lower fraction of the higher-order modes lades to the Kerr beam self-cleaning at 8 kW input peak-power in 5 m long few-mode fiber. With the larger transverse shift of the input beam, the number of propagating modes and the higher order modes fraction increase, and the peak-power for Kerr-beam self-cleaning also increases. In our experiments, we observed that in cases where the index of higher order modes is much more than that of fundamental mode and lower order modes, it is impossible to reach Kerr-beam self-cleaning. Therefore, in these fibers, there is a final limitation for the initial distribution and number of propagating modes to achieve a self-cleaned output beam.

In the second series of our experiments, we varied the tilt angle of the Gaussian laser beam at the input face of the few-mode step-index fiber. The incident angle was greater than the numerical aperture of the fundamental mode. In our selected coupling condition, the highest fraction of power has been coupled into the odds modes. As has been shown in GRIN fibers^26^, this modal distribution generates an off-axis refractive index modulation that leads to a strong overlap with the LP_11_ mode, and consequently, FWM processes with participation of this mode have the highest coefficient.

**Figure 2:**
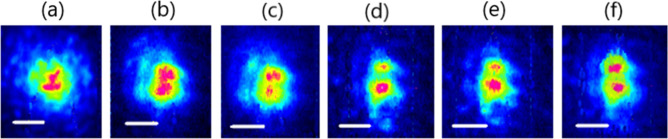
Near field intensity pattern at the few-mode step-index fiber output versus input peak power for appropriate input coupling conditions for higher fraction of LP_11_ mode, for (**a**) 1 kW, (**b**) 3.5 kW, (**c**) 5 kW, (**d**) 7.4 kW (**e**) 9.2 kW, (**f**) 12 kW. Scale bars 10 μm.

The experimental results for the Kerr-beam self-cleaning process in a 5 m long few-mode step-index fiber are shown in Fig. 2a–f. To confirm the experimental observation of self-cleaning to the LP_11_ mode in our considered fiber, we recorded the near field pattern of the output beam at different input peak-powers. By gradually increasing the injected power from 1kW in the linear propagation regime up to 12 kW in the nonlinear propagation regime, the main part of the launched power transfers toward the LP_11_ mode. The threshold peak-power to obtain Kerr beam self-cleaning in LP_11_ mode is on the order of 9–10 kW. This is a value greater than that for LP_01_ mode self-selection in step-index few-mode fiber (6 kW). To confirm the spatial reshaping toward LP_11_mode under special initial input conditions, we have investigated near-field and corresponding far-field images, which have been simultaneously observed in two cameras placed at the near and far fields, of the output beam from the step-index few-mode fiber in both the linear and nonlinear regimes.

**Figure 3:**
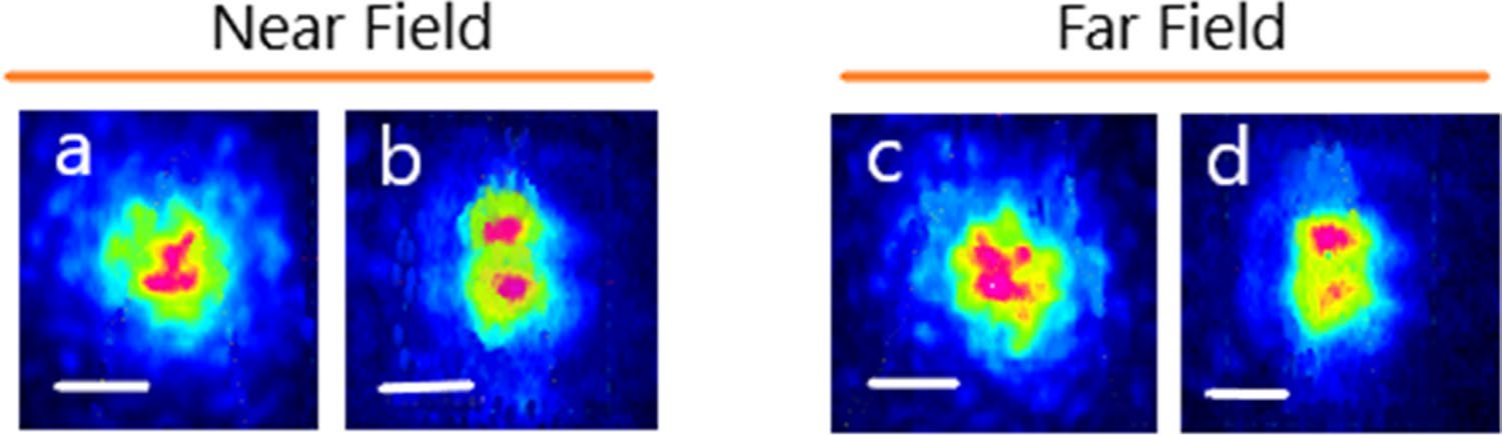
Near field (left) and far field (right) intensity patterns at the step-index few-mode fiber output for the linear propagation regime (**a**, **c**) and Kerr nonlinear regime (**b**, **d**) for appropriate input coupling conditions for a higher fraction of the LP_11_ mode, scale bars 10 μm.

**Figure 4:**
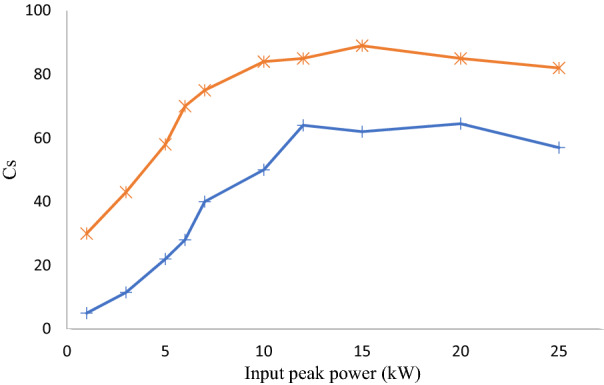
Intensity correlation C_S_ upon input peak power for LP_01_ (red curve) and LP_11_ (blue curve).

It is clear from Fig. 3 that nonlinear propagation for tilted input injection conditions leads to an apparent reshaping of both near and far field output profiles. It has been checked that there is no frequency conversion or Raman scattering at these power levels. We also show in Fig. 4 the intensity correlation C_s_ of the experimental near field output beam profile with the mode solver:

$$C_{s} = \frac{{\int {I_{exp} I_{th} ds} }}{{\sqrt {\int {I_{exp}^{2} ds} \int {I_{th}^{2} ds} } }}.$$

This represents the integration of the normalized product of the experimental output intensity profile ($I_{exp}$) and numerically calculated mode profile ($I_{th}$) on the fiber cross-section. The curves in Fig. 4 indicate that the intensity correlation for experimentally observed patterns corresponding to the LP_11_ and LP_01_ modes increases as the input peak power grows larger. Consequently, the power fraction into the LP_01_ and LP_11_ modes grows at high power levels.

## Numerical results

**Figure 5:**
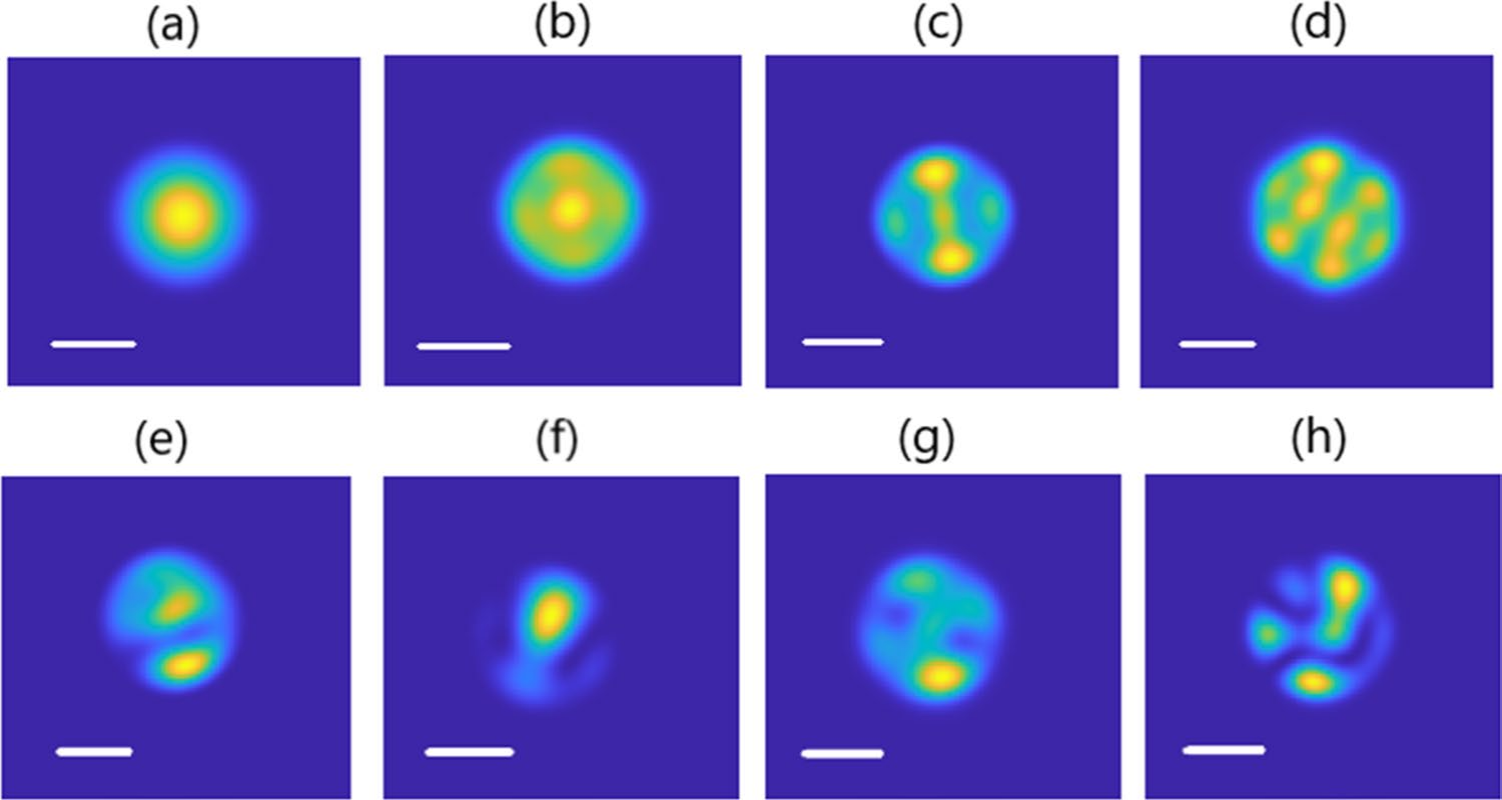
Numerical results of the output beam intensity patterns versus the number of excited modes at the input of the fiber for (**a**–**d**) 10 kW input peak-power in the nonlinear regime and (**e**–**h**) 0.1 kW input peak-power in the linear regime, scale bars 10 μm.

To guide our experiments, a series of numerical simulations has been performed by solving the generalized multimode nonlinear Schrödinger equation using an integration step of 0.05 mm and a transverse 800 × 800 grid for a spatial window of 100 × 100 μm. We considered a step-index MMF with a core diameter of 20 μm and a core-cladding index difference Δn = 0.0015 at a central wavelength 532 nm. Numerical results are consistent with the experimental results. Figure 5 summarizes a series of numerical simulations showing the output beam intensity patterns for different fractions of initially excited modes corresponding to different transverse shifts to the lateral position of the Gaussian beam with respect to the fiber core, for 8 kW input peak power in the nonlinear regime (Fig. 5a–d) and 0.1 kW input peak power in the linear regime (Fig. 5e–h), and for a 5 m length of the fiber. In these simulations, it has been considered that by increasing the displacement of the beam from the fiber center, the number of excited modes and also the fraction of higher order modes with respect to the central mode increased. Therefore, it can be seen in the nonlinear propagation regime that there is a bell-shaped output beam at the beginning, whereas by increasing the transverse shift, the spatial output shape of the beam will be disrupted. On the other hand, the modal distribution and population in the few-mode step-index fiber has a considerable effect on the spatial beam condensation effect. Receiving Kerr nonlinear self-cleaning at a higher number of excited modes needs to increase the input peakpower, where more nonlinear effects and wavelength broadening will be observed. Therefore, Kerr-beam self-cleaning in few-mode step index fibers can be seen for a limited number of initially excited modes. This is in agreement with our experimental results obtained by changing the input beam position with respect to the fiber center.

**Figure 6:**
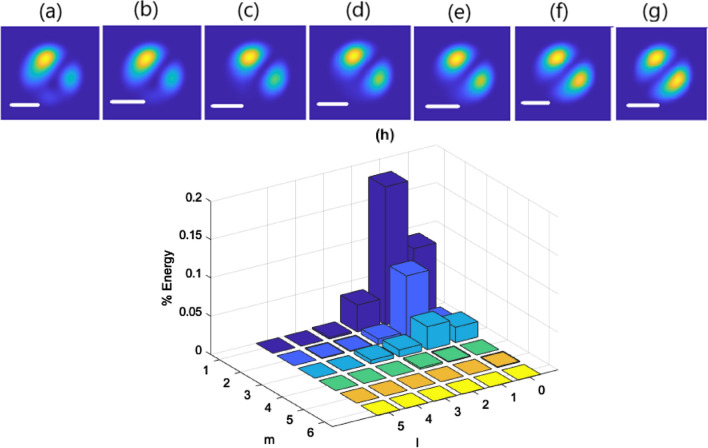
Numerical results of spatial reshaping of beam propagating in step-index few-mode fiber as a function of input peak power for (**a**) 0.5 kW, (**b**) 2 kW, (**c**) 4 kW, (**d**) 6 kW, (**e**) 7 kW, (**f**) 10 kW, (**g**) 13 kW, scale bars 10 µm. (**h**) Fraction of input power coupled into the different guided modes.

To investigate the possibility of Kerr nonlinear self-cleaning to LP_11_ mode in few-mode step-index fiber, in the next series of simulations, we have considered a different contribution of initial excited modes such that the highest fraction of power coupled to the LP_11_ mode, as shown in Fig. 6h. Under this condition, the numerical results of propagation of the input Gaussian beam in 5 m long fiber are consistent with the experimental observations. As the input peak power increases from 0.5 kW in the linear regime to 13 kW in the nonlinear regime, the spatial distribution of the output beam intensity transforms from a nearly multimode intensity pattern toward the LP_11_ mode spatial distribution (Fig. 6a–g). There are some slight differences between the experimental and corresponding numerical results of output beam intensity patterns for a particular input beam condition. This happens as a result of a little difference between laboratory coupling conditions and those we have considered in our simulations for any specific power of the input laser beam. But as it can be seen, the general process of the variations in beam intensity pattern by transformation from linear to nonlinear regime is exactly the same for experimental and numerical results. It should be noted that we didn’t observe any significant change in spectral width in our simulations.

## Conclusion

In conclusion, we experimentally demonstrate that Kerr nonlinear spatial reshaping of a pulsed beam to a nearly Gaussian mode at the output of a few-mode step-index fiber can be observed for specific distributions of initially excited modes. However, in the cases where the index of higher order modes is much more than that of fundamental mode and lower order modes, it is impossible to reach Kerr-beam self-cleaning. The initial distribution of guided modes affects the self-imaging distance, which has a significant rule on the Kerr-beam self-cleaning effect, and also a higher fraction of fundamental modes leads to irreversible decoupling of the central mode. Therefore, there is a final limitation for the initial coupling conditions and propagating mode numbers to achieve a condensed output beam pattern in few-mode step-index fibers.

We also demonstrated experimentally that Kerr nonlinear spatial cleaning can transform the output pattern into the LP_11_ mode of a few-mode step-index optical fiber while it has a speckled profile in linear regime. A necessary condition for nonlinear spatial reshaping into LP_11_ mode is to adjust the laser beam angle at the fiber input, which leads to a modal distribution in favor of the LP_11_ mode. Our numerical simulations are in agreement with experimental results. The observation of the possibility of output pattern engineering in step-index few-mode fibers may find practical importance in the delivery of high-power laser beams for a variety of applications including micromachining and nonlinear microscopy.

## References

[1] Forbes A (2014). Laser Beam Propagation: Generation and Propagation of Customized Light.

[2] Tzang O, Caravaca-Aguirre AM, Wagner K, Piestun R (2018). Adaptive wavefront shaping for controlling nonlinear multimode interactions in optical fibers. Nat. Photonics.

[3] Van Uden RGH, Amezcua Correa R, Antonio Lopez E, Huijskens FM, Xia C, Schülzgen G, Li A, de Waardt H, Koonen AMJ, Okonkwo CM (2014). Ultrahigh-density spatial division multiplexing with a few-mode multicore fiber. Nat. Photonics.

[4] Defienne H, Barbieri M, Walmsley IA, Smith BJ, Gigan S (2016). Two-photon quantum walk in amultimode fiber. Sci. Adv..

[5] Longhi S (2003). Modulational instability and space-time dynamics in nonlinear parabolic-index optical fibers. Opt. Lett..

[6] Aschieri P, Garnier J, Michel C, Doya V, Picozzi A (2011). Condensation and thermalization of classical optical waves in a waveguide. Phys. Rev. A.

[7] Pourbeyram H, Agrawal GP, Mafi A (2013). Stimulated Raman scattering cascade spanning the wavelength range of 523 to 1750 nm using a graded-index multimode optical fiber. Appl. Phys. Lett..

[8] Wright LG, Renninger WH, Christodoulides DN, Wise FW (2015). Spatiotemporal dynamics of multimodeoptical solitons. Opt. Express.

[9] Lopez-Galmiche G, Eznaveh ZS, Eftekhar MA, Antonio Lopez J, Wright LG, Wise F, Christodoulides D, Amezcua Correa R (2016). Visible supercontinuum generation in a graded index multimode fiber pumped at 1064 nm. Opt. Lett..

[10] Wright LG, Liu Z, Nolan DA, Li MJ, Christodoulides DN, Wise FW (2016). Self-organized instability ingraded index multimode fiber. Nat. Photonics.

[11] Koplow JP, Kliner DAV, Goldberg L (2000). Single-mode operation of a coiled multimode fiber amplifier. Opt. Lett..

[12] Krupa K, Tonello A, Shalaby BM, Fabert M, Barthelemy A, Millot G, Wabnitz S, Couderc V (2017). Spatial beam self-cleaning in multimode fibers. Nat. Photonics.

[13] Liu Z, Wright LG, Christodoulides DN, Wise FW (2016). Kerr self-cleaning of femtosecond-pulsed beams in graded-index multimode fiber. Opt. Lett..

[14] Podivilov EV, Kharenko DS, Gonta VA, Krupa K, Sidelnikov OS, Turitsyn S, Fedoruk MP, Babin SA, Wabnitz S (2019). Hydrodynamic 2D turbulence and spatial beam condensation in multimode optical fibers. Phys. Rev. Lett..

[15] Krupa K, Fona R, Tonello A, Labruyere A, Shalaby BA, Wabnitz S, Baronio F, Aceves AB, Millot G, Couderc V (2020). Spatial beam self-cleaning in second-harmonic generation. Sci. Rep..

[16] Leventoux Y, Parriaux A, Sidelnikov O, Granger G, Jossent M, Lavoute L, Gaponov D, Fabert M, Tonello A, Krupa K, Desfarges A, Kermene V, Millot G, Fevrier S, Wabnitz S, Couderc V (2020). Highly efficient few-mode spatial beamself-cleaning at 1.5 µm. Opt. Express.

[17] Krupa K, Tonello A, Couderc V, Barthélémy A, Millot G, Modotto D, Wabnitz S (2018). Spatiotemporal light-beam compression from nonlinear mode coupling. Phys. Rev. A.

[18] Moussa NO, Mansuryan T, Hage CH (2021). Spatiotemporal beam self-cleaning for high resolution nonlinear fluorescence imaging with multimode fiber. Sci. Rep..

[19] Guenard R, Krupa K, Dupiol R, Fabert M, Bendahmane A, Kermene V, Desfarges-Barthelemot A, Auguste JL, Tonello A, Barthlmy A, Millot G, Wabnitz S, Couderc V (2017). Kerr self-cleaning ofpulsed beam in ytterbium doped multimode fiber. Opt. Express.

[20] Mohammadzahery Z, Jandaghi M, Alipour S, SalimianRizi S, Hajinia E, Aghayari E, Nabavi H (2021). Nonlinear spatial reshaping of pulsed beam in a step-index few-mode optical fiber. Opt. Express.

[21] Krupa K, Tonello A, Barthélémy A, Mansuryan T, Couderc V, Millot G, Grelu P, Modotto D, Babin SA, Wabnitz S (2019). Multimode nonlinear fiber optics, a spatiotemporal avenue. APL Photonics.

[22] Zhu X, Schülzgen A, Li H, Li L, Han L, Moloney JV, Peyghambarian N (2008). Detailed investigation of self-imaging in large-core multimode optical fibers for application in fiber lasers and amplifiers. Opt. Express.

[23] Poletti F, Horak P (2008). Description of ultrashort pulse propagation in multimode optical fiber. Opt. Soc. Am. B.

[24] Li H, Brio M, Li L, Schuülzgen A, Peyghambarian N, Moloney JV (2007). Multimode interference in circular step-index fibers studied with the mode expansion approach. Opt. Soc. Am. B.

[25] Chen T, Zhang Q, Zhang Y, Li X, Zhang H, Xia W (2018). All-fiber passively mode-locked laser using nonlinear multimode interference of step-index multimode fiber. Photonics Res..

[26] Deliancourt E, Fabert M, Tonello A, Krupa K, Desfarges-Berthelemot A, Kermene V, Millot G, Barthlemy A, Wabnitz S, Couderc V (2019). Kerr beam self-cleaning on the LP_11_ mode in graded-index multimode fibers. OSA Continuum.

